# sRAGE in diabetic and non-diabetic critically ill patients: effects of intensive insulin therapy

**DOI:** 10.1186/cc10420

**Published:** 2011-08-26

**Authors:** Yaseen M Arabi, Mohammed Dehbi, Asgar H Rishu, Engin Baturcam, Salim H Kahoul, Riette J Brits, Brintha Naidu, Abderrezak Bouchama

**Affiliations:** 1 College of Medicine, King Saud bin Abdulaziz University for Health Sciences, Riyadh, 11426, Saudi Arabia; 2 Intensive Care Department, King Abdulaziz Medical City, Riyadh, 11426, Saudi Arabia; 3 Department of Biomedical Research, Biochemistry & Molecular Biology Unit, Dasman Diabetes Institute, 15462, Kuwait; 4 Experimental Medicine Department, King Abdullah International Medical Research Center, Riyadh, 11426, Saudi Arabia

## Abstract

**Introduction:**

Hyperglycemia represents an independent prognostic factor in critically ill non-diabetic patients but not in those with diabetes. In this context, there is an ongoing debate on the benefit of an intensive insulin therapy, particularly in diabetic patients. We tested the hypothesis that expression of the receptor for advanced glycation end-products (RAGE), an important signal transduction receptor that elicits long-lasting nuclear factor kappa B (NF-κB) activation, may underlie this difference. RAGE expression is regulated by multiple ligands, including high mobility group box-1 (HMGB-1), and is reflected by its released soluble form (sRAGE).

**Methods:**

A predesigned analysis was conducted of prospectively collected samples from 76 hyperglycemic critically ill patients (33 type-2 diabetes, 43 non-diabetes) aged ≥18 years with blood glucose of > 6.1 mmol/L enrolled in a randomized controlled trial comparing intensive insulin therapy with conventional insulin therapy. sRAGE and its ligand HMGB-1 together with IL-6, and soluble thrombomodulin (as markers of inflammation and endothelial cell injury, respectively) were evaluated in ICU, at Days 1, 3, 5 and 7. Plasma samples from 18 healthy subjects were used as controls.

**Results:**

Both diabetic and non-diabetic hyperglycemic patients showed increased plasma sRAGE, HMGB-1 and soluble thrombomodulin levels at the time of admission to ICU. Plasma IL-6 concentration was only increased in non-diabetic patients. Plasma levels of sRAGE were higher in diabetic compared with non-diabetic patients. Intensive insulin therapy resulted in a significant decrease of sRAGE and thrombomodulin at Day 7, in diabetic but not in non-diabetic patients. Circulating sRAGE levels correlated positively with IL-6 and soluble thrombomodulin levels and inversely with HMGB-1. Multivariate regression analysis demonstrated that sRAGE remains independently correlated with HMGB-1 only in diabetic patients. Neither sRAGE nor any inflammatory markers are associated with mortality.

****Conclusions**:**

These findings support the hypothesis that sRAGE release, time-course and response to intensive insulin therapy differ between hyperglycemic diabetic and non-diabetic critically ill patients. Whether this difference underlies the dissimilarity in clinical outcome of hyperglycemia in these two conditions warrants further studies.

## Introduction

Hyperglycemia represents an important independent risk factor for morbidity and mortality in critically ill patients admitted to ICU [1,2]. Accordingly, the benefit of strict control of blood sugar with intensive insulin therapy (IIT) versus conventional insulin therapy (CIT) has been greatly debated with some studies revealing benefit and others lack of benefit [3-6]. Moreover, the morbid consequences of hyperglycemia in critically ill patients, and the clinical effects of IIT, have been shown to differ according to diagnostic category [1-6]. In contrast to non-diabetic, hyperglycemia in ICU patients with type-2 diabetes is not independently associated with outcome, thus the benefit of IIT in this subpopulation remains unclear [1,2].

Hyperglycemia in the presence of oxidant stress promotes glycoxidation of intra- and extracellular proteins and an accumulation of advanced glycation end-products (AGE) [7]. Engagement of AGE with their receptor RAGE (a member of the immunoglobulin superfamily of cell-surface molecules), elicits activation of the transcription factor nuclear factor kappa B (NF-κB), expression of pro-inflammatory cytokines and induction of oxidative stress [7,8]. The RAGE-mediated inflammatory response has been implicated in micro- and macrovascular injury in diabetes [9,10]. Recent studies suggest that the pathogenic role of RAGE is not limited to diabetes but includes pathophysiological conditions characterized by excessive inflammatory response, such as sepsis and septic shock, acute lung injury, and intestinal dysfunction complicating hemorrhagic shock [11-17].

RAGE is activated by a large number of ligands including AGE, amyloid β-peptides, S-100 proteins and HMGB-1 [7]. Preventing ligand-RAGE interaction by the administration of recombinant soluble RAGE attenuated intestinal dysfunction in mouse models of resuscitated hemorrhagic shock and lipopolysaccharide (LPS)-induced lung injury [12,18]. RAGE knockout mice were shown to be resistant to septic shock induced by cecal ligation and puncture [11,16]. These observations suggest that the RAGE pathway is part of the systemic inflammatory response in critical illnesses and play a pathogenic role. Because the level of RAGE expression is regulated by the presence of its ligands, such as AGE, that are known to accumulate at a greater rate for a longer time in diabetic compared with non-diabetic patients, we hypothesized that the RAGE pathway may underlie the difference in the clinical effects of hyperglycemia as well as response to IIT between diabetic and non-diabetic patients admitted to ICU [19].

RAGE has several soluble receptor isoforms resulting from alternative splicing of the full length mRNA or proteolysis of the cell-surface receptor [20,21]. An increased circulating total pool of sRAGE reflects enhanced tissue expression of RAGE in type 2-diabetic and non-diabetic subjects [22,23]. Hence, using an assay that measures the extracellular domain of sRAGE, we examined whether there is differential release of plasma sRAGE between hyperglycemic diabetic and non-diabetic critically ill patients admitted to ICU. Furthermore, the effects of conventional and intensive insulin therapy on the time course of sRAGE expression and the release of HMGB-1, thrombomodulin, and IL-6 were evaluated.

## Materials and methods

### Study population

The present investigation included 76 hyperglycemic critically ill (33 type-2 diabetes, 43 non-diabetes) consecutive patients who stayed at least three days in the ICU. Diagnosis of diabetes was based on the history as established by the patient primary team prior to hospital admission. The study population was part of a randomized controlled study that evaluated the effects of IIT versus CIT on the outcome of 523 critically ill patients. Informed consent was obtained from all patients included in the study before randomization. Insulin therapy was titrated to maintain blood glucose concentrations between 4.4 and 6.1 mmol/L in the IIT group and 10 and 11.1 mmol/L in the CIT group [6]. Patients were eligible in the original study, if they were ≥18 years and were hyperglycemic (blood glucose of > 6.1 mmol/L during the first 24 hours of ICU admission). They were not included if they had type I diabetes, diabetic ketoacidosis, documented hypoglycemia on ICU admission or in the same hospitalization, brain death, do-not-resuscitate status, terminal illness defined as expected survival of less than four weeks as judged by the treating physician, post cardiac arrest, seizures within past six months, pregnancy, liver transplantation, burn victims, readmission to ICU within the same hospitalization, expected ICU length of stay (LOS) of < 24 hrs, inability to obtain consent within the randomization window of 24 hrs of ICU admission, and enrollment in a competing trial, as published elsewhere [6]. The frequency of blood glucose monitoring was once hourly and was increased to every 20 minutes when blood glucose levels decreased to ≤3.2 mmol/L, then was reduced to every two to four hours when measurements were at the target level. The study was approved by the Institutional Review Board of the King Abdulaziz Medical City.

### Blood sampling

Blood samples were collected in EDTA-treated tubes on the day of admission to ICU, then on Days 3, 5 and 7. The samples were immediately centrifuged at 4°C for 20 minutes at 1,600 g. Plasma samples were stored at -80°C until assayed.

### Measurement of sRAGE, HMGB-1, thrombomodulin and IL-6 plasma levels

Plasma concentrations of sRAGE, HMGB-1, thrombomodulin and IL-6 were measured in accordance with the manufacturers' instructions using commercial ELISA kits from R&D Systems (Minneapolis, MN, USA (Quantikine human IL-6 and sRAGE)), Diagnostica Stago (Asnieres, France (Asserachrom thrombomodulin)) and Shino-test Corporation (Tokyo, Japan (HMGB-1)). Eighteen plasma samples from healthy individuals were included in the sRAGE, HMGB-1, thrombomodulin and IL-6 assays.

### Statistical analysis

Descriptive variables are given as the mean ± SE. Skewed data are presented as median and interquartile ranges (IQR 25th to 75th percentile). Comparisons were performed using Wilcoxon and Kruskal-Wallis tests. The mixed linear model was used to compare the groups over time. Pearson correlation coefficients were used to determine the degree of the linear relationship between the continuous variables. Linear and logistic regression analyses were performed to determine the associations between outcome, inflammatory markers and clinical and biochemical variables. A multivariate linear regression model was used to test the interaction between renal function assessed by the calculated creatinine clearance and diabetes on plasma sRAGE levels. Differences were considered significant at *P *< 0.05. The statistical analysis of data was done by using the statistical software package SAS version 9.1.3 (Statistical Analysis System, SAS Institute Inc., Cary, NC, USA).

## Results

### Clinical characteristics of the study population

Table 1 shows that diabetic patients are significantly older, with higher body mass index, blood sugar concentration and severity of illness and lower creatinine clearance on admission than non-diabetics. Hospital (but not ICU) mortality is also significantly higher in diabetic than non-diabetic patients. However, the present study was not powered to evaluate clinical outcomes.

**Table 1:** Baseline characteristics and outcome of diabetic and non-diabetic patients admitted in ICU

	Diabetic		Non-Diabetic		
	**IIT** **(*n *= 16)**	**CIT** **(*n *= 17)**	**IIT** **(*n *= 27)**	**CIT** **(*n *= 16)**	***P***
**Age (years)**	66.1 ± 3.5	65.9 ± 2.0	49.3 ± 4.5	43.4 ± 5.2	< 0.0001
**Sex, male: female**	6:10	7: 10	22:5	13:3	0.0001
**BMI**	31.2 ± 1.6	33.8 ± 2.6	25.1 ± 0.8	26.6 ± 1.0	0.0003
**APACHE II score**	28.6 ± 1.9	29.4 ± 1.7	22.3 ± 1.6	24.1 ± 2.0	0.001
**Blood glucose (mmol/L)**	15.0 ± 1.1	13.4 ± 0.9	11.1 ± 0.7	10.0 ± 0.4	< .0001
**Creatinine (μmol/l)**	178 ± 31	222 ± 47	119 ± 20	144 ± 41	0.14
**Creatinine clearance (ml/min)**	55 ± 10	52 ± 9	90 ± 9	95 ± 15	0.007
**Admission category**					
**Medical**	16	16	21	14	0.15
**Surgical**	0	1	6	2	0.15
**SOFA score (Day 1)**	9.3 ± 0.7	10.9 ± 0.8	8.3 ± 0.6	10.0 ± 0.7	0.11
**SOFA score (Day 3)** **SEPSIS**	7.4 ± 3.0	8.6 ± 3.7	7.0 ± 4.0	8.9 ± 2.6	0.25
**ICU acquired sepsis**	9	7	11	8	0.89
**Length of hospital stay (days)**	64.4 ± 14.4	42.6 ± 8.8	48.4 ± 10.0	48.3 ± 9.5	0.77
**ICU mortality, no**.	4	3	6	2	0.84

**Hospital mortality, no**.	**9**	**11**	**7**	**3**	0.001

Values represent mean (SE) in age, BMI, Acute Physiologic Score and Chronic Health Evaluation (APACHE II) score, blood glucose, and Sequential Organ Failure Assessment (SOFA) score on admission to ICU. *P-*values represent the comparison between diabetic (*n *= 33) and non-diabetic patients (*n *= 43). Creatinine clearance = (140 - age(yr))*weight(kg))/(72*serum Cr(mg/dL)) (multiply by 0.85 for women) [34].

### Circulating levels sRAGE, HMGB-1, thrombomodulin and IL-6 in study population

**Figure 1:**
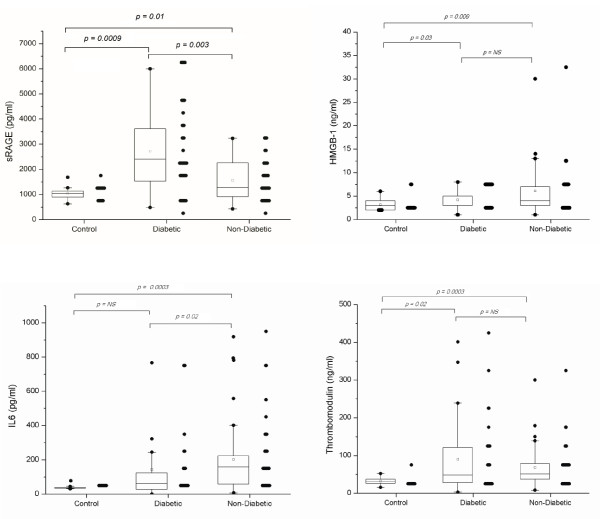
**Plasma sRAGE, HMGB-1, thrombomodulin and IL-6 in diabetic and non-diabetics and control upon admission**.

On the day of admission to ICU, plasma sRAGE, HMGB-1 and thrombomodulin levels are significantly higher in all critically ill patients with or without diabetes as compared with healthy control subjects (Figure 1). Plasma IL-6 levels are significantly higher in non-diabetic but not in diabetic patients as compared with values from healthy control (Figure 1).

### Circulating levels sRAGE, HMGB-1, thrombomodulin and IL-6 in diabetic and non-diabetic patients

**Figure 2:**
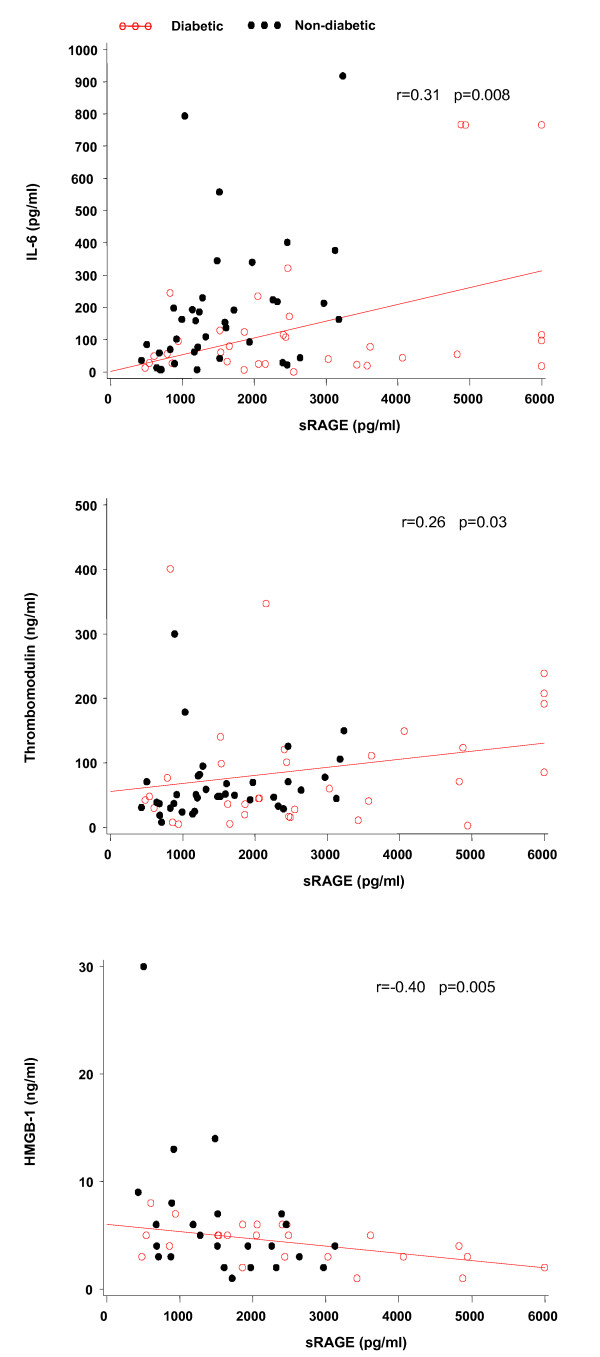
**Correlation between plasma sRAGE, HMGB-1, soluble thrombomodulin and IL-6, respectively**. r represents the coefficient correlation.

Figure 1 shows that the plasma sRAGE concentration is significantly higher and IL-6 lower when diabetic patients are compared with those without diabetes (median (IQR) 2,406 (1,534 to 3,613) vs. 1,302 (918 to 2,260 pg/ml); *P *= 0.003) and (61 (27 to 124) vs. 159 (59 to 224 pg/ml); *P *= 0.02), respectively. No differences in HMGB-1 and soluble thrombomodulin is found between these two groups of patients. Plasma sRAGE levels correlate positively with plasma IL-6 and soluble thrombomodulin levels and inversely with those of HMGB-1 (Figure 2).

### Effects of insulin therapy on sRAGE, HMGB-1, thrombomodulin and IL-6 in diabetic and non-diabetic patients

**Figure 3:**
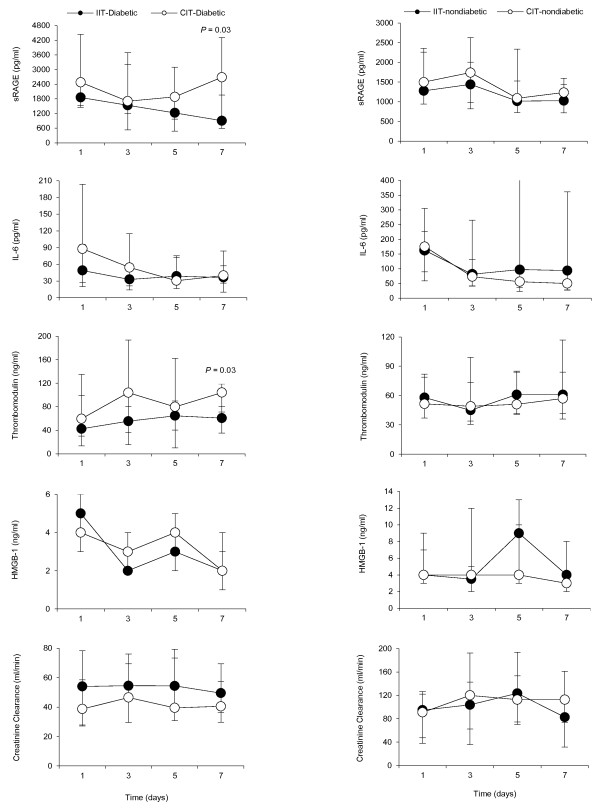
**Effects of insulin therapy on sRAGE, IL-6, thrombomodulin, HMGB-1 and creatinine clearance in diabetic and non-diabetics**.

On the day of admission to ICU (day 1), the plasma levels of sRAGE, HMGB-1, thrombomodulin and IL-6 are similar between IIT and CIT in diabetic and non-diabetic patients (Figure 3). Compared with conventional insulin therapy, IIT does not influence the time course of sRAGE, HMGB-1, thrombomodulin and IL-6 in non-diabetic patients (Figure 3). However, in patients with diabetes, IIT significantly decreases plasma sRAGE (903 (586 to 2,732) vs. 2,684 (1,956 to 4,312) pg/ml, *P *= 0.03) and thrombomodulin (61 (35 to 81) vs. 104 ng/ml, *P *= 0.03) at day 7 post-admission, while creatinine clearance did not significantly change over time (Figure 3).

### Interrelationship between sRAGE, biochemical markers and outcome in diabetic and non-diabetic patients

Multivariate regression analysis demonstrates that sRAGE remains independently correlated with HMGB-1 only in diabetic patients (Table 2). Neither levels of blood glucose and plasma creatinine, nor age, body mass index (BMI), Acute Physiology and Chronic Health Evaluation (APACHE) II score, creatinine clearance, and Sequential Organ Failure Assessment (SOFA) score are independently associated with sRAGE (Table 2). No significant interaction between the creatinine clearance and diabetes on plasma sRAGE levels was found (*P *= 0.08).

**Table 2:** Univariate and multivariate linear regression analyses of factors associated with sRAGE in diabetic and non-diabetics

	Diabetic				Non-diabetic			
	Univariate		Multivariate		Univariate		Multivariate	
*Variable*	*Estimate*	*P*	*Estimate*	*P*	*Estimate*	*P*	*Estimate*	*P*
Age (years)	-10.991	NS			-4.456	NS		
Gender	769.540	NS			88.840	NS		
BMI	-4.315	NS			-42.630	NS		
APACHE II score	89.353	0.03	63.82	NS	28.739	NS		
SOFA score	186.919	NS			60.331	NS		
IL-6 (pg/ml)	3.479	0.01	1.22	NS	1.486	0.01	1.68	NS
Thrombomodulin (ng/ml)	4.165	NS			2.248	NS		
HMGB-1 (ng/ml) *	-544.690	0.001	-509.97	0.002	-56.697	0.03	-49.56	0.059
Creatinine (μmol/l)	1.834	NS			2.658	0.005	1.58	NS
Creatinine clearance (ml/minute)	-6.957	NS			-2.999	NS		
Blood glucose (mmol/L)	-37.363	NS			-21.311	NS		
PaO_2_/FIO_2 _(mm Hg)	0.638	NS			1.066	NS		
INR	441.567	NS			16.071	NS		

* *P *< 0.05 for both uni-and multivariate analysis

APACHE, Acute Physiologic Score and Chronic Health Evaluation; BMI, body mass index; HMGB-1, high mobility group box-1; IL-6, interleukin-6; INR, international normalized ratio; PaO_2_/FIO_2, _ratio of partial pressure of oxygen to the fraction of inspired oxygen; SOFA, Sequential Organ Failure Assessment.

Uni- and multivariate logistic regression analyses demonstrate that hyperglycemia is an independent predictor of mortality in non-diabetic patients but not in patients with diabetes. Neither sRAGE nor any inflammatory markers are associated with mortality (Table 3). However, these findings should be interpreted cautiously due to the small sample size and the large heterogeneity of the study groups.

**Table 3:** Univariate and multivariate logistic regression analysis of factors associated with mortality in diabetic and non-diabetics

	Diabetic				Non-diabetic			
	Univariate		Multivariate		Univariate		Multivariate	
*Variable*	*Estimate*	*P*	*Estimate*	*P*	*Estimate*	*P*	*Estimate*	*P*
Age (years)	0.012	NS			0.036	0.04		NS
Gender	0.464	NS			0.875	NS		
BMI	-0.075	NS			-0.083	NS		
APACHE II score	0.100	NS			0.124	0.02		NS
SOFA score *	0.345	0.02	0.357	0.03	0.009	NS		
sRAGE (pg/ml)	0.0004	NS			-0.00008	NS		
IL-6 (pg/ml)	0.00003	NS			0.001	NS		
Thrombomodulin (ng/ml)	0.004	NS			0.007	NS		
HMGB-1 (ng/ml)	-0.321	NS			0.046	NS		
Creatinine (μmol/L)	-0.002	NS			0.003	NS		
Creatinine clearance (ml/minute)	-0.0096	NS			-0.028	0.01	-0.058	NS
Blood glucose (mmol/L)*	0.196	NS			0.514	0.003		0.03
PaO_2_/FiO_2 _(mm Hg)	0.0009	NS			-0.006	NS		
INR *	2.753	0.01	2.328	0.04	3.176	0.02		NS

* *P *< 0.05 for both uni-and multivariate analysis

APACHE, Acute Physiologic Score and Chronic Health Evaluation; HMGB-1, high mobility group box-1; IL-6, interleukin-6; INR, international normalized ratio; PaO_2_/FIO_2_, ratio of partial pressure of oxygen to the fraction of inspired oxygen; SOFA, Sequential Organ Failure Assessment; sRAGE, soluble receptor for advanced glycation end-products.

## Discussion

Findings from this study reveal that on admission to ICU, critically ill patients with hyperglycemia display elevated circulating levels of sRAGE, HMGB-1 and soluble thrombomodulin. Circulating plasma IL-6 levels were increased only in non-diabetic patients. This suggest that sRAGE is a component of the host's systemic inflammatory response to hyperglycemic critical illness.

More important, the results show that diabetic and non-diabetic patients display distinct sRAGE release and time course pattern to acute illness as well as response to IIT. The plasma sRAGE concentration is significantly higher in diabetic patients than in those without diabetes. Administration of IIT attenuates significantly the levels of sRAGE at ICU day 7 only in diabetic patients. These findings suggest albeit indirectly that RAGE pathway activation and response to IIT differ between hyperglycemic diabetic and non-diabetic critically ill patients. Whether this accounts for the difference in clinical course and outcome observed between these two conditions could not be ascertained because of the discrepancy in baseline characteristics between the two study groups. Nonetheless, the findings warrant further investigations because of their potential clinical and therapeutic implications. Indeed, if different mechanistic pathways of inflammation are at play in hyperglycemic diabetic and non-diabetic critically ill patients, the use of a uniform therapeutic approach for all patients may not be physiologically sound.

RAGE is a pattern recognition receptor that has been shown to contribute to both initiation and perpetuation of inflammation in experimental sepsis [7,11,12,14,16]. In contrast to experimental studies, there is a paucity of clinical data [24-26]. In healthy human volunteers, LPS administration leads to increased plasma sRAGE levels [24]. In patients with sepsis or septic shock, plasma sRAGE was found elevated during the first 24 hours in ICU, with the highest levels in non-survivors [26]. In another study, plasma sRAGE was associated with outcomes in patients with acute lung injury ventilated with higher tidal volumes [17]. Findings in the present study are in accordance with these reports by showing that sRAGE levels are elevated upon admission to ICU. It also extends them by demonstrating that the rise of plasma sRAGE levels remain sustained over a week and are not independently associated with outcome. Whether these long-lasting levels of plasma sRAGE reflect persistent RAGE pathway activation and thereby contribute to the perpetuation of inflammation in critically ill patients merit further studies.

Several studies have examined the association between sRAGE levels and renal function, and revealed that sRAGE is independently associated with decreased glomerular filtration rate [27-29], although the mechanisms underlying this relationship are not well understood. Both increased plasma sRAGE levels merely due to reduced elimination by failing kidneys, as well as a result of RAGE up-regulation to protect against AGE-accumulation and induction of tissue damage have been postulated. Most of these studies were confined to elderly stable patients in chronic renal failure with or without diabetes [27-29]. More recently, in patients with severe traumatic injury, plasma levels of sRAGE were found to predict the development of acute renal failure [30]. Taken together, these observations have prompted us to examine the association between sRAGE levels and renal function in our patients and the extent to which it could explain the observed difference in plasma sRAGE levels between the diabetic and non-diabetic patients. Using a multivariate linear regression model, interaction of diabetes with renal function could not be demonstrated, which means that the difference in plasma sRAGE levels on admission between diabetic and non-diabetic patients could not be explained on the basis of difference in renal function. Although, this finding should be interpreted with caution due to the small sample size of our study population, it may suggest that circulating sRAGE levels in hyperglycemic critically ill diabetic and non-diabetic patients reflect RAGE up-regulation. Although, the cellular source and precise role of sRAGE in our patients remain unclear, sRAGE has been demonstrated to exert a protective effect by working as a decoy domain receptor averting ligands binding to RAGE and, thereby, preventing inflammatory injury [7,31]. In this context, we determined that plasma HMGB-1 levels, a RAGE-ligand, and plasma IL-6 levels as a marker of systemic inflammation were increased in our study population. Surprisingly, we found that although in more severe conditions on admission to ICU, several patients with diabetes displayed a subdued inflammatory response as assessed by circulating IL-6 levels compared with non-diabetic patients. This finding is intriguing because there are very scarce data on the systemic inflammatory response to critical illness in diabetic patients that result in ICU admission. Nonetheless, earlier study demonstrated that the basal production of IL-6 and TNF-α in cultured diabetic blood was markedly depressed in comparison with non-diabetic samples [32]. The release of IL-6 and TNF-α increased following stimulation with LPS, but IL-6 remained significantly lower in diabetic patients than in controls, thus lending support to our observation [32]. The findings in the present study also reveal that plasma sRAGE level is independently correlated with HMGB-1 in diabetic patients only. Hence, it is appealing to speculate that sRAGE may have fulfilled its function as decoy in binding HMGB-1 in diabetic patients. Whether this explains the attenuated inflammatory response in this condition is unclear and merits further study.

Previous study demonstrated that IIT protects the endothelium of critically ill patients by down-regulating iNOS gene expression; however, diabetic and non-diabetic patients were not segregated [33]. Indeed, the present findings agree with this observation by showing that both diabetic and non-diabetic critically ill patients exhibit endothelial cell activation/injury as reflected by increased levels of soluble thrombomodulin. The study also reveals that IIT accelerates the decline of soluble thrombomodulin levels, suggesting a comparable protective effect of the endothelium but only in diabetic patients. Further, the concomitant decrease of sRAGE with thrombomodulin raises a novel hypothesis that IIT attenuated endothelial injury via RAGE down-regulation in this subpopulation.

Some limitations of this study merit consideration. First, the 76 patients studied comprise a small subgroup from a total sample of 523 in the medial-surgical ICU. Considering the inclusion and exclusion criteria of the RCT, the results may not be extended to all hyperglycemic critically ill patients whether they are diabetic or not. Second, the immunoassay used in this study discriminates neither the RAGE that results from cleavage of the cell-surface receptor nor the different sRAGE splice variants [20]. Indeed, sRAGE isoforms have been shown to vary among cell type, and to display different affinity for RAGE ligands [20]. In our study, the cellular source of sRAGE was unclear, thus, for future studies specific assays for each splice variant would be required for investigating the significance of sRAGE in clinical samples.

## Conclusions

Despite the limitations, the current study has revealed that sRAGE release, time-course and response to IIT differ between hyperglycemic diabetic and non-diabetic critically ill patients. Whether this difference underlies in part the dissimilarity in the immune response and outcome to critical illness in these two conditions warrants further studies.

## Key messages

• Both diabetic and non-diabetic hyperglycemic patients display increased plasma sRAGE, HMGB-1 and soluble thrombomodulin levels at the time of ICU admission

• Plasma levels of sRAGE are higher in critically ill diabetic patients compared with hyperglycemic non-diabetic patients

• Intensive insulin therapy significantly decreases plasma sRAGE and soluble thrombomodulin in diabetic patients, but not in those without diabetes

• RAGE pathway activation and response to IIT differ between hyperglycemic diabetic and non-diabetic critically ill patients

## Abbreviations

AGE: advanced glycation end-products; APACHE II: Acute Physiology and Chronic Health Evaluation; BMI: body mass index; CIT: conventional insulin therapy; EDTA: ethylene diamine tetra acetic acid; HMGB-1: high mobility group box-1; IIT: intensive insulin therapy; IL-6: interleukin-6; iNOS: inducible form of nitric oxide synthase; IQR: interquartile range; LOS: length of stay; LPS: lipopolysaccharide; mRNA: messenger RNA; NF-κB: nuclear factor kappa B; RAGE: receptor for advanced glycation end-products; RCT: randomized controlled trial; SAS: Statistical Analysis System; SOFA: Sequential Organ Failure Assessment; TNF: tumor necrosis factor.

## Competing interests

The authors declare that they have no competing interests.

## Authors' contributions

The PI (YMA) had full access to all of the data in the study and takes responsibility for the integrity of the data and the accuracy of the data analysis. YMA, AHR and AB were responsible for the conception and design. YMA, AHR, EB, SHK, RJB and BN took part in the acquisition of data. EB, SHK, RJB and BN were involved in blood sample collection. AB, EB, SHK, RJB and BN were involved in laboratory work. YMA, MD and AB were responsible for analysis and interpretation of data. YMA, MD and AB were responsible for drafting the manuscript. YMA, AHR, EB and AB were in charge for the critical revision of the manuscript. YMA, MD and AB were responsible for statistical analysis. YMA, AHR and AB were in charge for the general supervision.
